# Novel Methods for Managing and Assessing Gait and Posture in Pediatric Population

**DOI:** 10.3390/children10060976

**Published:** 2023-05-31

**Authors:** Shashank Ghai

**Affiliations:** 1 Department of Political, Historical, Religious and Cultural Studies, Karlstad University, 651 88 Karlstad, Sweden; shashank.ghai@kau.se; 2 Centre for Societal Risk Research, Karlstad University, 651 88 Karlstad, Sweden

The ability to walk and maintain proper posture is fundamental to human mobility and independence. It is especially important for children as it forms the basis for their ability to move. However, there are various obstacles that can hinder children from achieving optimal gait and posture, such as developmental delays, neurological disorders, musculoskeletal conditions, and injuries. Addressing these problems is vital because impaired walking and posture can profoundly affect a child’s physical, psychological, and social well-being. Additionally, it can lead to secondary complications like muscle imbalances, joint deformities, cardiovascular issues, and more, ultimately diminishing their quality of life [1]. To improve gait and posture of children, it is crucial to create novel interventions that meet their unique needs [2]. While traditional approaches like physical therapy have shown effectiveness, there is growing recognition that more innovative and personalized approaches are necessary [3]. This special edition presents a variety of articles that further our understanding of novel assessments and interventions for improving gait and posture in the pediatric population. The special issue encompasses a case study [4], two case series [5,6], a prospective case-controlled study [7], and an updated meta-analysis [8], providing a comprehensive overview of the topic.

In a case series by Sassi, Faccioli [5], the authors demonstrated the importance of novel gait and posture assessment strategies for determining appropriate rehabilitative interventions. The authors discussed two rare cases of Progressive Pseudohematoid Dysplasia in siblings manifesting gait abnormalities and muscle weakness. The authors performed a comprehensive analysis, including spatiotemporal, kinematic, and kinetic gait assessments, along with surface electromyography. The analysis indicated a decrease in various spatiotemporal aspects of gait, such as reduced gait speed and stride length. Likewise, the kinematic analysis revealed impairments in the hip, knee, and ankle joints, while kinetic analysis of the vertical ground reaction forces revealed a significant reduction in ankle power during push-off. These findings were supported by surface electromyography, which showed a considerable decrease in gastrocnemius activity. Building upon these findings, the authors proposed innovative rehabilitative strategies, including motion observation, motor imagery, and dynamic ankle foot orthoses as potential methods for enhancing the children’s motor abilities and overall quality of life.

Similarly Panzeri, Genova [6], demonstrated the importance of personalized rehabilitation interventions in their pilot study as the authors examined the impact of visual biofeedback training in a virtual reality setting. The researchers investigated the effects of 20 individualized training sessions, combined with physical therapy on gait performance, walking endurance, and gross motor function of six children and teenagers with acquired brain injury and hemiparesis. The intervention involved delivering multisensory feedback tailored to each individual’s needs, including visual, proprioceptive, and acoustic cues. For example, visual feedback exercises were employed to enhance dorsiflexor activation in a hemiparetic child with foot drop caused by hypoactivation of dorsiflexors, whereas kinematic biofeedback-based training was prescribed for another child with impaired knee movement during the swing and stance phase of gait. All six participants demonstrated clinical improvements aligned with the researchers’ goals of enhancing gait performance, gross motor function, or walking endurance through personalized training. The authors highlighted the significance of early intervention, as one of their participants with the shortest time since injury exhibited the greatest clinical improvement compared to another participant who underwent training after 74 months of injury.

In another case report by Frade, Neves [4], the authors reported the beneficial influence of early intervention and personalized training approaches in a unilateral neonatal brachial plexus injury affecting the C5-C6 roots. Although this article did not directly address the impact of an intervention on gait and postural outcomes, it introduced innovative transdisciplinary interventions and important care models that were relevant to the current issue. The authors described a conservative rehabilitation approach involving various techniques, such as mobilizations, electrical stimulations, Kinesio taping, constrained-induced movement therapy, splinting, massage, botulinum toxin, and stretching. These interventions aimed to restore function and enhance sensorimotor development in the affected limb of the child. The authors also highlighted the significance of early intervention in this case, not only for facilitating limb development but also for preventing the onset of potential neuromuscular complications. Furthermore, the authors emphasized a unique collaborative model of care involving both clinicians and the child’s parents. According to the authors, this collaborative model enabled parents to replicate the rehabilitative interventions at home, thereby complementing and enhancing the effectiveness of the intervention.

Moreover, Ghai, Ghai [8] in an updated meta-analysis, re-iterated the predominant influence of personalized and multisensory interventions for enhancing gait and postural outcomes in individuals with cerebral palsy. The authors updated the findings of a previous review [9] by examining 14 studies and confirmed significant improvements in gait speed and stride length through their meta-analysis, both within and between groups. They also found that auditory stimulations positively impacted gait deviation index and gross motor function for standing and walking, with varying degrees of improvement. An interesting finding from the study was the comparison between auditory stimulation-based training and no training. The authors reported that training with auditory stimulation was superior as compared to the transient application of auditory stimulations. According to the authors, this training may have facilitated the coordination between auditory and motor network nodes, establishing a connected interfaced map between them [10]. The authors also proposed several personalized and tailored approaches for applying auditory stimulations in pediatric gait rehabilitation. For instance, the authors suggested sonification feedback, which translates kinematic joint parameters into acoustic signal characteristics. They also recommended future studies to integrate multisensory interventions, such as incorporating tactile stimulation via taping alongside auditory stimulation. Finally, in a prospective, case-controlled study by Herdea, Neculai [7], the authors demonstrated the potential benefits of a minimally invasive surgical intervention on gait and quality of life outcomes in a pediatric population group with flexible flatfoot. Specifically, the authors assessed the long-term consequences of a minimally invasive subtalar arthroereisis surgery on 33 children with bilateral flexible pes planovalgus. During a two-year follow-up, the authors revealed that the surgical intervention significantly improved the children’s post-operative quality of life. Notably, this improvement was comparable to a healthy control group of 36 children. The authors also observed a significant decrease in the average running time and an improvement in foot aesthetics after the surgery.

In conclusion, this special issue presents novel assessment methods and interventions for improving gait and postural outcomes in the pediatric population. It is important to note that the sample sizes of the included studies are small, making generalizability of the results challenging. Nonetheless, these findings present innovative approaches and pave the way for future studies to further evaluate these interventions’ effects on gait and postural outcomes in children.
